# HNF1*α* Controls Liver Lipid Metabolism and Insulin Resistance via Negatively Regulating the SOCS-3-STAT3 Signaling Pathway

**DOI:** 10.1155/2019/5483946

**Published:** 2019-05-15

**Authors:** Jiaorong Tan, Jiahong Xu, Guohua Wei, Lijuan Zhang, Long'e Sun, Guangyu Wang, Fei Li, Fengxiang Jiang

**Affiliations:** ^1^Department of Endocrinology, People's Hospital of Shanghai Putuo, Tongji University School of Medicine, Shanghai 200060, China; ^2^Department of Cardiology, Tongji Hospital, Tongji University School of Medicine, Shanghai 200065, China; ^3^Department of Gastroenterology, People's Hospital of Shanghai Putuo, Tongji University School of Medicine, Shanghai 200060, China

## Abstract

This study is aimed at evaluating the effects, functions, and mechanism of HNF1*α* on hepatic glycolipid metabolism. In this study, free fatty acid- (FFA-) induced steatosis of hepatocyte liver cell LO2 was used as an *in vitro* model. The methods of Oil Red O staining, RT-qPCR, western blot, and immunofluorescence staining were used to detect LO2-regulated HNF1*α* expression and its effects on FFA-induced LO2 cell steatosis, the insulin signaling and SOCS-3-STAT3 signaling pathways, the expression of lipid metabolism-related regulators, and phosphorylation. With increased FFA induction time, the expression of HNF1*α* in the LO2 fatty degeneration hepatic cells gradually decreased. Downregulation of HNF1*α* expression aggravated FFA-induced steatosis of LO2 hepatocytes. HNF1*α* promotes activation of the insulin pathway and oxidative breakdown of fat and inhibits lipid anabolism. Inhibitors of STAT3 can reverse the regulation of decreased HNF1*α* expression on the insulin signaling pathway and fat metabolism. We also confirmed this pathway using HNF1*α*-/- mice combining treatment with STAT3 inhibitor NSC 74859 *in vivo*. HNF1*α* regulates hepatic lipid metabolism by promoting the expression of SOCS-3 and negatively regulating the STAT3 signaling pathway.

## 1. Introduction

Nonalcoholic fatty liver disease (NAFLD) refers to a type of chronic liver disease characterized by excessive deposition of fat in hepatocytes that is not due to alcohol or other defined liver factors [1–6]. The liver is an important metabolic organ: after the food is degraded into glucose, fatty acids, and amino acids by the gastrointestinal tract, these products reach the liver through blood circulation where they are metabolized to provide energy for normal functioning. If liver metabolism is abnormal, it can cause harm to the body. In patients with NAFLD, excessive deposition of fat in liver cells not only affects the progression of other chronic liver diseases but may also lead to serious liver diseases such as cirrhosis and hepatocellular carcinoma. This increased understanding of the harmfulness of NAFLD has led some researchers to question whether it is correctly classified as benign lesions [7, 8]. NAFLD is not only inextricably linked to the development of many liver diseases but also closely related to “metabolic syndromes” such as obesity and insulin resistance. Insulin resistance leads to a decrease in the efficiency of cellular uptake and utilization of glucose, resulting in a disorder of cellular glycolipid metabolism. Previous results showed that NAFLD is closely related to insulin resistance, which increases the risk of type 2 diabetes [9, 10]. Given the close relationship between NAFLD, insulin resistance, and diabetes, the main components of metabolic syndrome, NAFLD is now commonly considered to be an important early warning signal for liver manifestations and metabolic syndromes. NAFLD is extremely harmful and has a high incidence. A meta-analysis showed that the prevalence of NAFLD is about 25% worldwide and about 27% in Asia [11]. With the increase in high-sugar and high-fat diets, the prevalence of NAFLD has shown a clear upward trend. It is possible that in the near future, NAFLD will become a severe disease worldwide.

Hepatocyte nuclear factor 1*α* (HNF1*α*) is a transcription factor mainly expressed in liver tissues, where it regulates the expression of multiple liver-specific genes and plays an important role in maintaining normal liver function. Mutations in the HNF1*α* gene have been found in rare cases of hepatocellular adenomas, rare benign liver tumors, and noncirrhotic hepatocellular carcinomas [12]. In addition to liver tissue, HNF1*α* is also expressed in the pancreas and kidneys. Mutations in the HNF1*α* gene cause functional defects in islet *β* cells and reduced insulin secretion, leading to maternal onset diabetes of the young 3 (MODY3) [13]. Previous work showed that lipid metabolism in patients with MODY3 differs from that of patients with type 2 diabetes and nondiabetic patients [14]. Patients with MODY3 also have elevated bile acid synthesis [15]. Double knockdown of the HNF1*α* gene in mice causes multiple symptoms such as hepatomegaly, phenylketonuria, Fanconi syndrome, and noninsulin-dependent diabetes mellitus [16]. In summary, HNF1*α* is involved in multiple metabolic pathways which play an important role in maintaining normal metabolism of the body. However, its regulation mechanism is still unclear.

Deletion of HNF1*α* leads to increased secretion of inflammatory factors [17, 18]. Chronic inflammation, especially visceral obesity, contributes to the development of metabolic diseases [19–21]. Many inflammatory factors are known to be involved in signal transduction by activating the STAT3 signaling pathway. The STAT3 signaling pathway functions in cell proliferation, differentiation, apoptosis, and immune regulation and thus is essential to maintaining the normal function of cells. However, the STAT3 signaling pathway is strictly regulated. SOCS3 is one of the important negative feedback regulators of the STAT3 signaling pathway. The effects of inflammatory factors or chronic inflammatory responses on metabolic-related diseases such as NAFLD are associated with sustained activation of the STAT3 signaling pathway. Thus, the STAT3 signaling pathway is closely related to metabolic regulation and metabolism.

In this study, FFA-induced steatosis LO2 hepatocytes were used as an *in vitro* model to evaluate both the regulation of HNF1*α* on hepatic lipid metabolism and the relationship between the HNF1*α* and SOCS3-STAT3 signaling pathways. Our results provide both a powerful theoretical basis and new potential drug targets for the regulation of HNF1*α* on hepatic lipid metabolism and treatment of nonalcoholic fatty liver.

## 2. Materials and Methods

### 2.1. Mouse Studies

C57/BL6 male mice were purchased from Shanghai Laboratory Animal Company (SLAC, Shanghai, China). HNF1a−/− mice were obtained from Charles River Laboratories and heterozygous mice were mated to obtain homozygous mutant mice as reported [22]; 8-week-old male mice were fed with either NC (protein 18.3%, fat 10.2%, carbohydrates 71.5%, D12450B, Research Diets) or an HFD (protein 18.1%, fat 61.6%, carbohydrates 20.3%, D12492, Research Diets) ad libitum for up to 8 weeks. Started at the 5th week, indicated groups of mice were given NSC 74859 (Selleck), 5 mg/kg, i.v. every 2 days for 5 doses. All animal procedures were approved by the Institutional Animal Care and Use Committee at People's Hospital of Shanghai Putuo, Tongji University School of Medicine. Mice were sacrificed at the 8th week, and the livers were taken for weight, Oil red O staining, or further analysis. Serum was collected for biochemical assays.

### 2.2. Culture of LO2 Cells

LO2 cells were purchased from The Cell Bank of Type Culture Collection of Chinese Academy of Sciences (Shanghai Institute of Cell Biology, Chinese Academy of Sciences, Shanghai, China). Cells were cultivated in RPMI-1640 medium (Hyclone, 11875093) supplemented with 10% fetal bovine serum (Gibco, 10099141), 10,000 U/mL penicillin, and 0.1 mg/mL streptomycin (Sigma, V900920) at 5% CO_2_, 37°C.

### 2.3. Free Fatty Acid Induction of Lipolysis in LO2 Cells

The FFA solution was prepared by mixing 0.5 mM oleic acid (Sigma, O7501) and 0.25 mM palmitic acid (Sigma, P9767), LO2 cells were treated will FFA solution to induce steatosis in LO2 cells, and steatosis was detected at 12, 24, 36, and 48 h after the FFA treatment.

### 2.4. Oil Red O Staining

LO2 cells were fixed with 4% paraformaldehyde for 10 min. After staining for 30 min with 60% Oil Red O (Sigma, O0625) isopropanol solution, they were washed with 60% isopropanol, and cell steatosis was observed under a microscope (Olympus). Frozen liver sections (4 *μ*m) were stained with Oil Red O (Sigma) for 30 minutes. The sections were counterstained with Mayer hematoxylin after destaining in 60% isopropanol.

### 2.5. Biochemical Assays

The contents of the LO2 cells and liver tissue triglyceride (TG) (Sigma, MAK266), cholesterol (TC) (Sigma, MAK043), and nonesterified fatty acid (NEFA) (Sigma, MAK044) were determined by using the corresponding kits and manufacturer's instructions using a microplate reader (Thermo Scientific). Serum glucose levels were measured with a glucometer (One Touch Ultra Easy, Life Scan). Serum fasting insulin was measured by ELISA (Millipore). The homeostasis model assessment of the IR index was calculated as HOMA − IR = [FBG (mmol/l) × FIns (mIU/l)]/22.5.

### 2.6. RT-PCR Detection of mRNA Levels

Total RNA was extracted by using TRIzol (Invitrogen), and the first strand of cDNA was reverse transcribed using a reverse transcription kit (Takara, 639522). Real-time quantitative PCR was performed using a PCR instrument (Bio-Rad) with the GAPDH as an internal reference using the One Step SYBR® PrimeScript™ RT-PCR Kit (Takara, RR066A). The primer sequences for HNF1*α* fragment amplification are 5′-AACACCTCAACAAGGGCACTC-3′ and 5′-CCCCACTTGAAACGGTTCCT-3′, the primer sequences for SREBP-1c fragment amplification are 5′-ATCGGCGCGGAAGCTGTCGGGGTAGCGTC-3′ and 5′-ACTGTCTTGGTTGTTGATGAGCTGGAGCAT-3′, the primer sequences for PPAR fragment amplification are 5′-CAAGTGCCTTTCTGTCGG-3′ and 5′-TGTTTCCATCTTCGCTGT-3′, and the primer sequences for GAPDH internal reference are 5′-ACAACTTTGGTATCGTGGAAGG-3′ and 5′-GCCATCACGCCACAGTTTC-3′.

### 2.7. WB Detection

The total cellular or liver tissue protein was obtained by lysing the cells with RIPA lysate (Sigma, V900854), and the protein concentration was determined using a BCA protein concentration assay kit (Sigma, FP0010). Equal amounts of protein were electrophoresed on a 10% Bis-Tris gel at 120 V for 1 h, the protein was transferred to the PVDF membrane at 350 mA for 70 minutes, and the PVDF membrane was blocked with 5% BSA in TBST buffer for 1 h. The primary antibody was incubated by gentle shaking at 4°C overnight, and the secondary antibody was incubated for 1 h at room temperature. ECL hypersensitive luminescent solution (Thermo, 32132) was used for color reaction, and gray scale was detected by image laboratory software (Bio-Rad) to quantitatively analyze protein expression. The antibodies used included HNF1*α* antibody (Abcam, ab96777), IRS-1 antibody (CST, #2382), phospho-IRS-1 antibody (CST, #2385), AKT antibody (CST, #9272), phospho-Akt antibody (CST, #4060), SOCS3 antibody (CST, #2932), STAT3 antibody (CST, #9139), phospho-STAT3 (CST, #9134), SREBP1 antibody (Abcam, ab191857), and PPAR*α* antibody (Abcam, ab8934). The GAPDH protein antibody (Abcam, ab8245) was selected as an internal reference.

### 2.8. Immunofluorescence Staining

Cells were fixed with 4% paraformaldehyde (Sigma, 158127) for 10 min, permeated with 0.5% Triton X-100 in PBS for 20 min at room temperature, and then, the cells were blocked with 5% BSA for 1 h. HNF1*α* antibody (Abcam, ab96777) was added and incubated overnight at 4°C. Fluorescent secondary antibody (Abcam, ab150077) was added for 2 h at room temperature. Cells were then incubated with DAPI for 10 min at room temperature and observed using a fluorescence microscope (Nikon).

### 2.9. HNF1*α* Overexpression and Knockdown Vector Construction

A 1893 bp HNF1*α* cDNA fragment was obtained by RT-PCR and cloned into the pcDNA3.1 vector (addgene) to construct an HNF1*α* expression vector. The shRNA sequence CCGGAGACTGCAGAAGTACCCTCAACTCGAGTTGAGGGTACTTCTGCAGTCTTTTTTTG was inserted into the pGPU6/Neo vector (GenePharma, Shanghai, China) to construct an HNF1*α* RNAi interference vector.

### 2.10. Data Analysis

Statistical analysis was performed using SPSS16.0 software, using the Student *t*-test with *p* < 0.05 considered a significant difference.

## 3. Results

### 3.1. FFA Induces the Degeneration of LO2 Cells to Decrease the Expression of HNF1*α*

**Figure 1:**
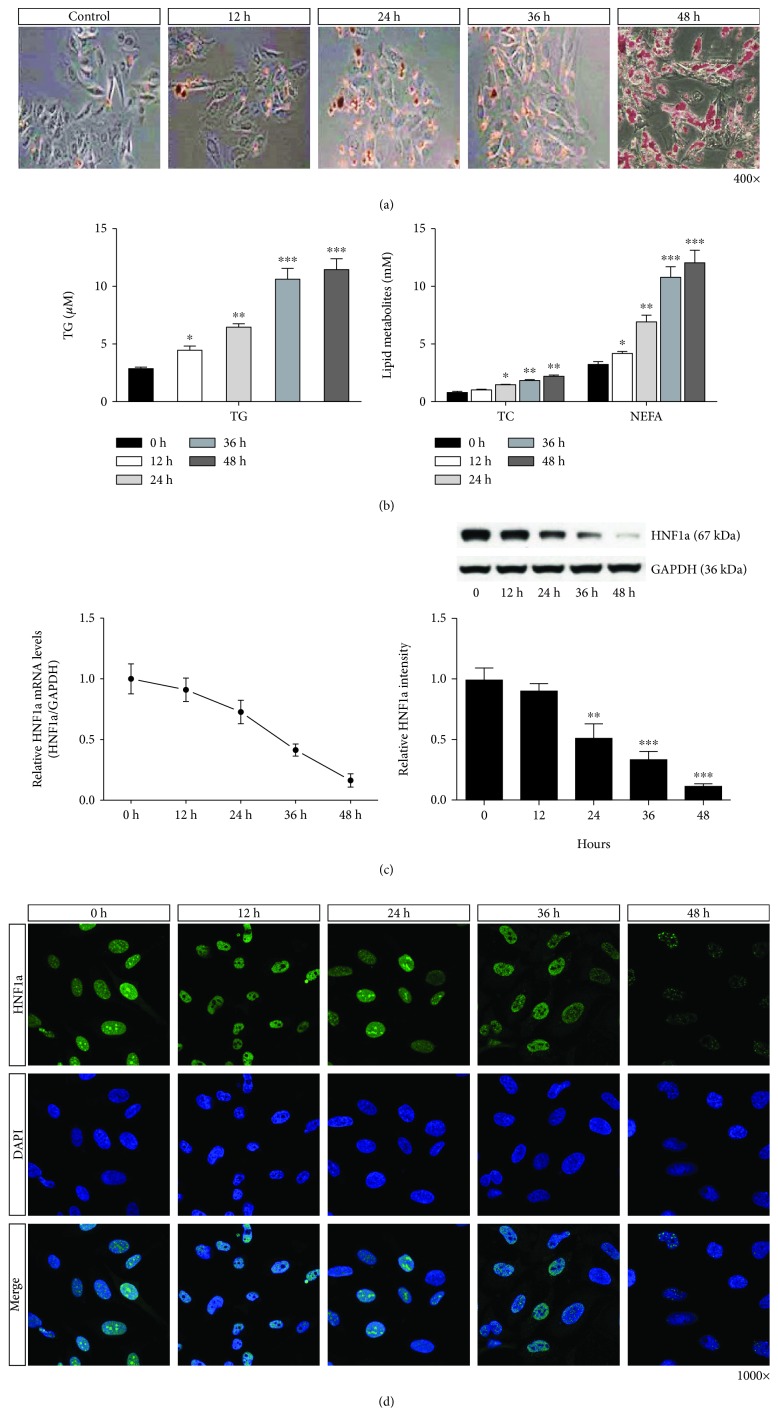
Decreased HNF1*α* expression in FFA-induced fatty degeneration of LO2 cells. (a) Oil Red O staining showed that the number of lipid droplets in LO2 cells increased gradually with increased FFA induction time. (b) Biochemical indicators showed that with increasing FFA induction time, TC, TG, and NEFA content in the LO2 cells increased gradually. Reported values are the means + SD of the three independent tests, with ^∗^*p* < 0.05, ^∗∗^*p* < 0.01, and ^∗∗∗^*p* < 0.001. (c, d) RT-qPCR, western blot, and immunofluorescence staining showed that the expression of HNF1a mRNA and protein decreased with increasing FFA induction time.

The results of Oil Red O staining showed that the amount of red granular lipid droplets in LO2 cells gradually increased and the fatty degeneration gradually increased with the extension of the time for the FFA treatment (Figure 1(a)). The results of biochemical indicators showed that the total cholesterol (TG), triglyceride (TC), and nonesterified fatty acid (NEFA) levels of LO2 cells showed a significant upward trend with increasing FFA treatment time (Figure 1(b)). The results of RT-qPCR, western blot, and fluorescent immunoassay showed that the fatty degeneration of LO2 cells increased with FFA treatment time, and the mRNA and protein expression of HNF1*α* showed a significant downward trend (Figures 1(c) and 1(d)).

### 3.2. HNF1*α* Inhibits FFA-Induced Fatty Degeneration in LO2 Cells

**Figure 2:**
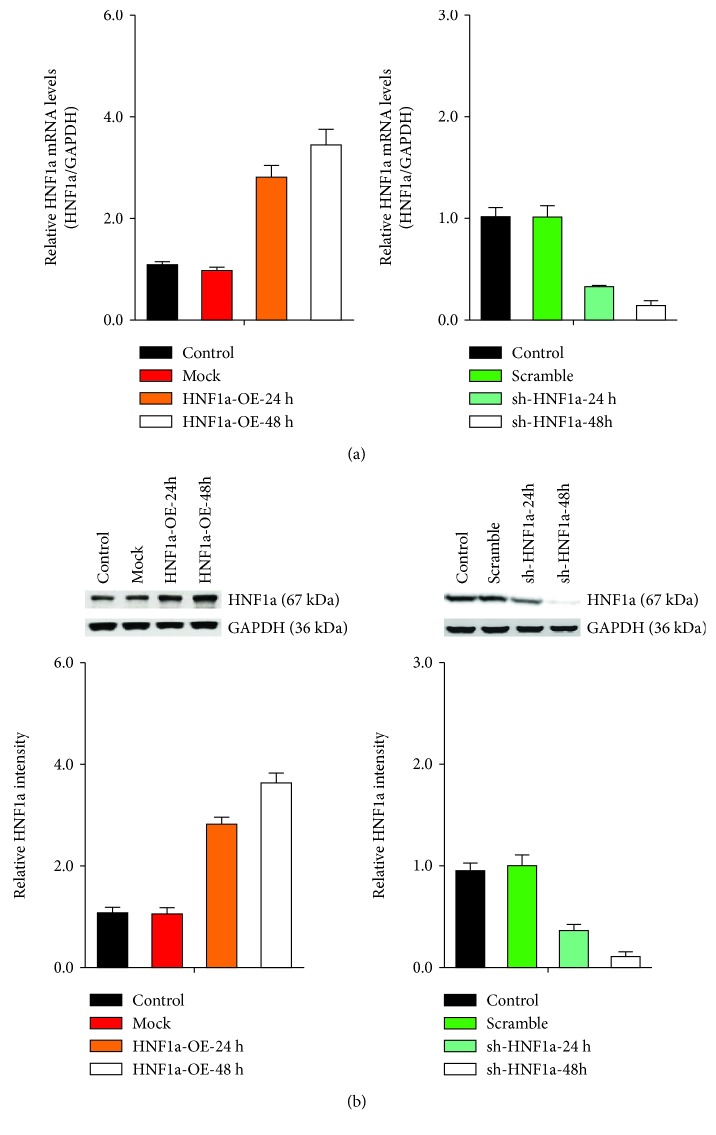
Efficiency assay for the HNF1*α* overexpression and knockdown vector. (a) RT-qPCR showed that the gene expression of HNF1*α* was effectively regulated in the overexpression and knockdown transgenic plants. (b) Western blot analysis showed that the HNF1*α* protein was upregulated in the overexpression lines and downregulated in the knockdown lines.

To determine whether HNF1*α* affects FFA-induced steatosis of hepatocyte LO2, overexpression vectors and shRNA knockdown vectors were used to regulate HNF1*α* expression. All of the above vectors are capable of efficiently regulating mRNA and protein expression levels (Figures 2(a) and 2(b)).

**Figure 3:**
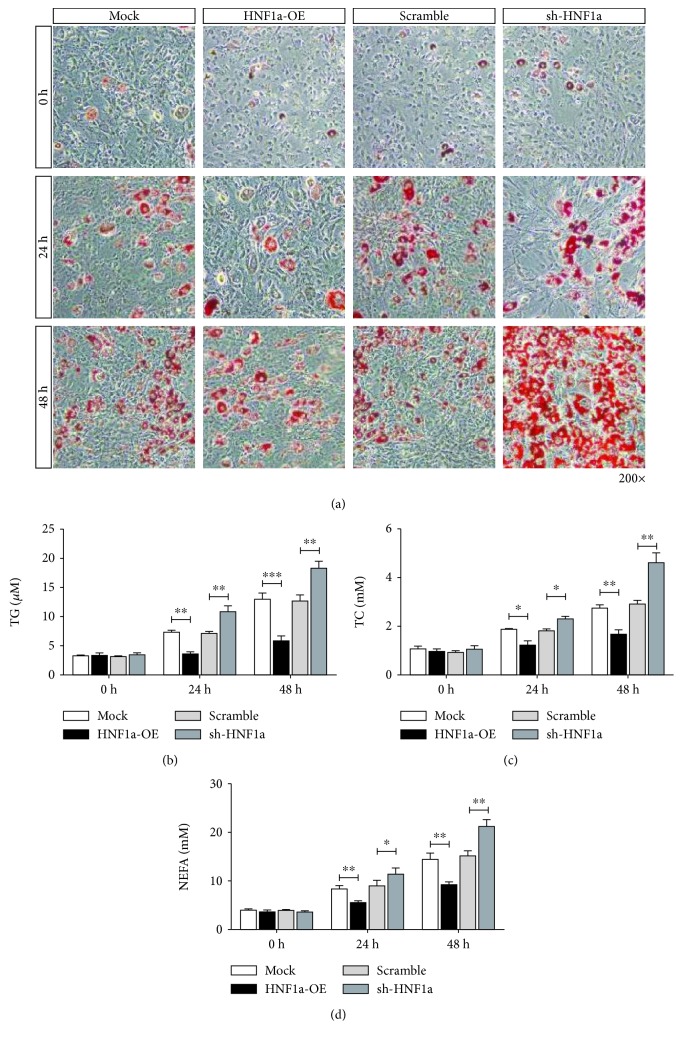
HNF1*α* inhibits FFA-induced steatosis in LO2 cells. (a) Oil Red O staining showed that the number of red granular lipid droplets decreased after upregulating the expression of HNF1*α* and the number of red granular lipid droplets increased after downregulating the expression of HNF1*α*. (b–d) Biochemical indicator tests showed that the upregulation of HNF1*α* expression caused significant decreases in TC, TG, and NEFA content. Downregulation of HNF1*α* expression led to significantly increased content of TC, TG, and NEFA. Reported values are the means + SD of the three independent tests, with ^∗^*p* < 0.05, ^∗∗^*p* < 0.01, and ^∗∗∗^*p* < 0.001.

The results of Oil Red O staining showed that after upregulating the expression of HNF1*α*, the number of red granular lipid droplets in LFA-induced LO2 cells decreased and steatosis was reduced when compared to the control. By downregulating the expression of HNF1*α*, FFA induction increased the number of red granular lipid droplets in LO2 cells and aggravated fatty degeneration (Figure 3(a)). Compared with the control, upregulation of HNF1*α* expression in the FFA-induced LO2 cells significantly decreased the content of TG, TC, and NEFA, while downregulation increased them (Figures 3(b)–3(d)).

### 3.3. HNF1*α* Promotes Activation of the Insulin Signaling Pathway

**Figure 4:**
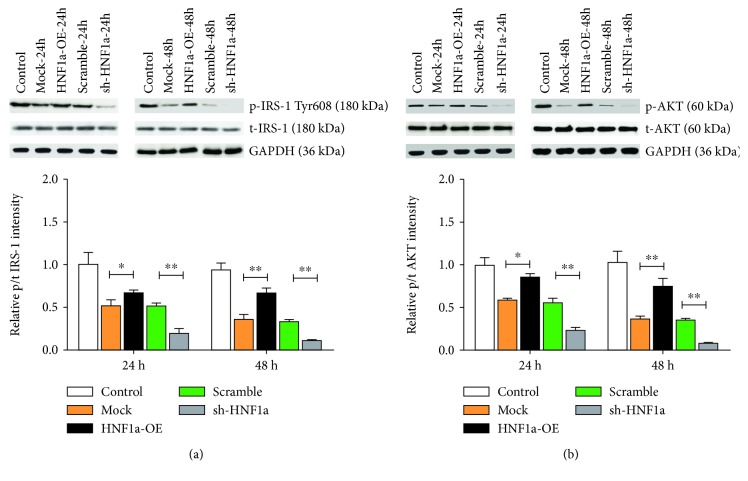
HNF1*α* promotes activation of the islet signaling pathway. (a, b) Western blot analysis showed that upregulation of HNF1*α* expression promoted IRS-1 and AKT phosphorylation and downregulation of HNF1*α* expression inhibited IRS-1 and AKT phosphorylation. Reported values are the means + SD of the three independent tests, with ^∗^*p* < 0.05, ^∗∗^*p* < 0.01, and ^∗∗∗^*p* < 0.001.

In order to evaluate the effect of HNF1*α* on the insulin signaling pathway, two important regulatory factors, IRS-1 and AKT, were selected as representatives in this pathway. Western blot analysis showed that FFA-induced phosphorylation of IRS-1 and AKT in LO2 cells was significantly increased after the upregulation of HNF1*α* expression, whereas FFA-induced IRS-1 and AKT in LO2 cells were downregulated after the HNF1*α* expression was downregulated. The phosphorylated water decreased significantly on average (Figures 4(a) and 4(b)).

### 3.4. HNF1*α* Inhibits the STAT3 Pathway, Promotes Lipolytic Metabolism, and Inhibits Lipid Anabolism

**Figure 5:**
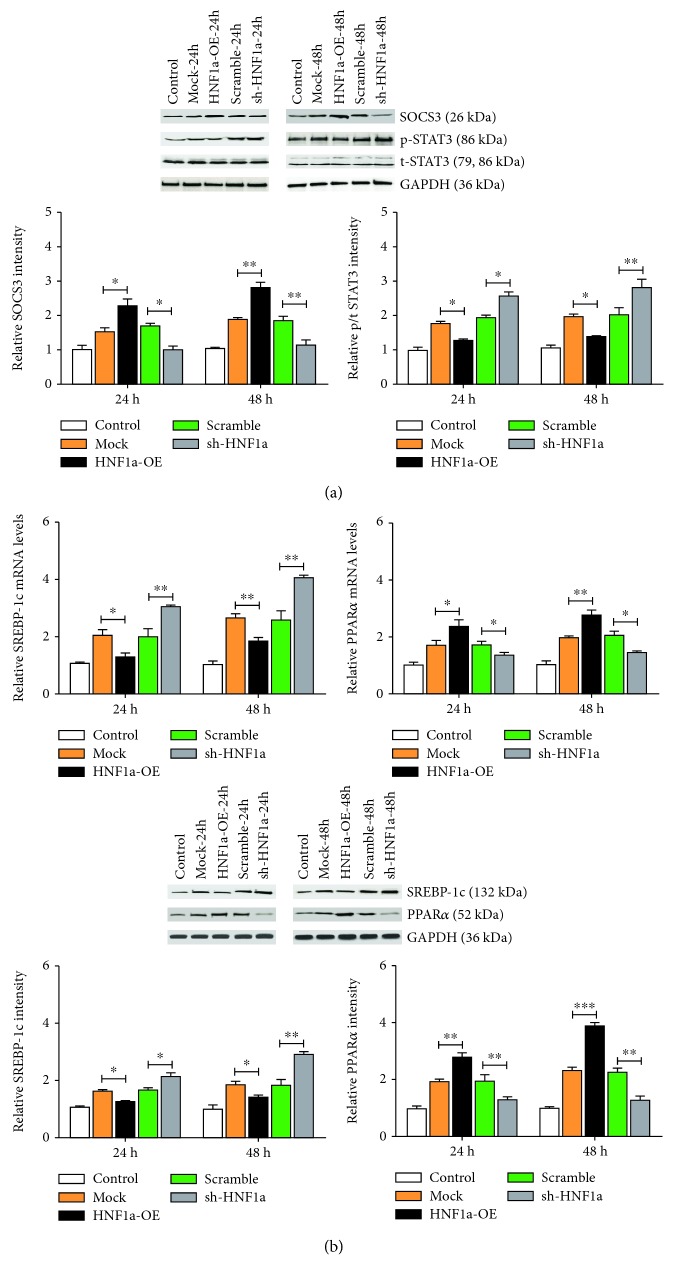
HNF1*α* inhibits the STAT3 pathway and promotes lipolytic catabolism lipid anabolism. (a) Western blot analysis showed that upregulation of HNF1*α* expression promoted the expression of SOCS-3, inhibited the phosphorylation of STAT3, downregulated the expression of HNF1*α*, inhibited the expression of SOCS-3, and promoted the phosphorylation of STAT3. (b) RT-qPCR and western blot showed that upregulation of HNF1*α* expression inhibited the expression of SREBP-1c, promoted the expression of PPAR*α*, downregulated the expression of HNF1*α*, promoted the expression of SREBP-1c, and inhibited the expression of PPAR*α*. Reported values are the means + SD of the three independent tests, with ^∗^*p* < 0.05, ^∗∗^*p* < 0.01, and ^∗∗∗^*p* < 0.001.

To investigate the association between the HNF1*α* and STAT3 signaling pathways, we examined the expression of HNF1*α*, SOCS3, and STAT3 and phosphorylation at 24 h and 48 h. Western blot analysis showed that upregulation of HNF1*α* expression promoted FCS-induced SOCS3 expression in LO2 cells and inhibited STAT3 phosphorylation, whereas downregulation of HNF1*α* expression inhibited FFA-induced LOCS cell SOCS3 expression and promoted STAT3 phosphorylation (Figure 5(a)).

In order to evaluate the effect of HNF1*α* on lipid metabolism, we examined the expression of HNF1*α* and mRNA and protein expression of SREBP-1c and PPAR*α* by RT-qPCR and western blot at 24 h and 48 h. Compared with the control group, the expression of SREBP-1c mRNA and protein in LOF cells decreased significantly with increasing expression of HNF1*α* and the expression of PPAR*α* mRNA and protein increased significantly. With a decrease in HNF1*α* expression, mRNA and protein levels of SREBP-1c in FFA-induced LO2 cells increased significantly and PPAR*α* mRNA and protein expression decreased significantly (Figure 5(b)).

### 3.5. STAT3 Inhibitor NSC74859 Can Reverse the Effect of the Downregulation of HNF1*α* Expression on Hepatic Lipid Metabolism

In the current study, we found that HNF1*α* regulates the SOCS3-STAT3 signaling pathway. Together with previous studies, we hypothesized that the effect of HNF1*α* on hepatic metabolism may be achieved through the SOCS3-STAT3 signaling pathway. To confirm this inference, NSC74859, the inhibitor of STAT3, was used.

**Figure 6:**
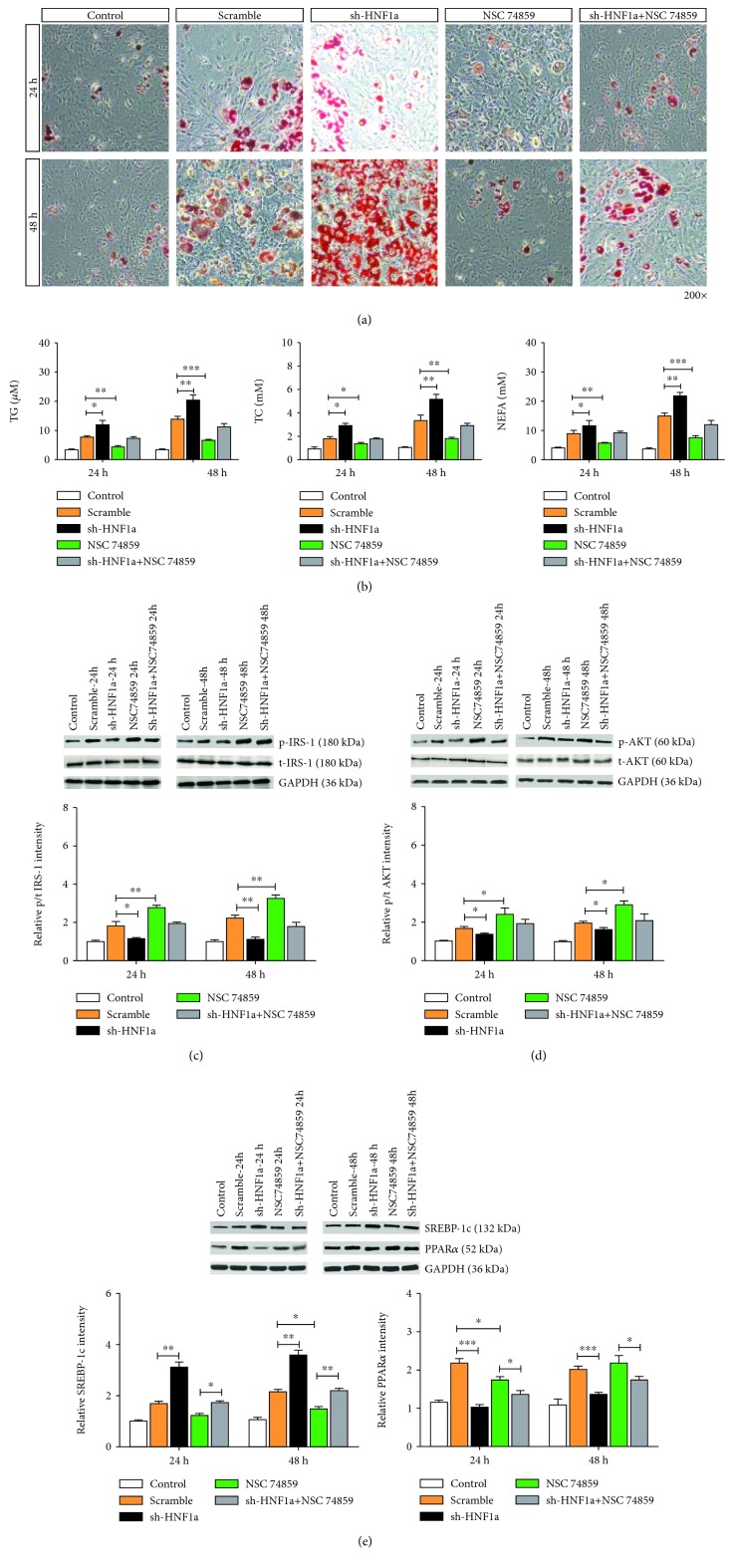
STAT3 inhibitor NSC74859 can reverse the effect of the downregulation of HNF1*α* expression on hepatic glycolipid metabolism. (a) Oil Red O staining test showed that NSC74859 reduced the number of red granular lipid droplets in LO2 cells induced by FFA. Downregulation of HNF1*α* expression followed by addition of NSC74859 reduced FAR-induced red granule lipid droplets in LO2 cells. (b) Biochemical indicators showed that NSC74859 reduced TC, TG, and NEFA contents. After downregulating the HNF1*α* expression and adding NSC74859, the contents of TC, TG, and NEFA decreased significantly. (c, d) Western blot analysis showed that NSC74859 promoted IRS-1 and AKT phosphorylation. Downregulation of HNF1*α* expression followed by addition of NSC74859 abolished the inhibition of IRS-1 and AKT phosphorylation by downregulating the HNF1*α* expression. (e) Western blot analysis showed that NSC74859 inhibited the expression of SREBP-1c and promoted the expression of PPAR*α*. Downregulation of HNF1*α* expression followed by NSC74859 reversed the effects of downregulation of HNF1*α* expression on SREBP-1c and PPAR*α* expression. Reported values are the means + SD of the three independent tests, with ^∗^*p* < 0.05, ^∗∗^*p* < 0.01, and ^∗∗∗^*p* < 0.001.

The Oil Red O staining test showed that the addition of 100 nM NSC74859 reduced the number of red granular lipid droplets in the FFA-induced LO2 cells and alleviated the steatosis of LO2 cells. Adding NSC74859 after the downregulation of HNF1*α* expression reversed the increase in the number of red granular lipid droplets in LO2 cells induced by FFA downregulation of HNF1*α* expression, aggravating the phenomenon of steatosis in LO2 cells (Figure 6(a)). The biochemical results showed that NSC74859 significantly decreased the levels of TG, TC, and NEFA in FFA-induced LO2 cells. The addition of NSC74859 also significantly reduced the levels of TG, TC, and NEFA that were elevated by downregulating the HNF1*α* expression (Figure 6(b)). Western blot analysis showed that NSC74859 promoted phosphorylation of IRS-1 and AKT (Figures 6(c) and 6(d)). NSC74859 can somewhat alleviate the inhibition of IRS-1 and AKT phosphorylation caused by downregulating the HNF1*α* expression. NSC74859 inhibits the expression of SREBP-1c and promotes the expression of PPAR*α*. NSC74859 reversed the effects of downregulation of HNF1*α* expression on SREBP-1c and PPAR*α* expression (Figure 6(e)).

### 3.6. HNF1*α* Inhibits Steatosis through Suppressing STAT3 *In Vivo*

**Figure 7:**
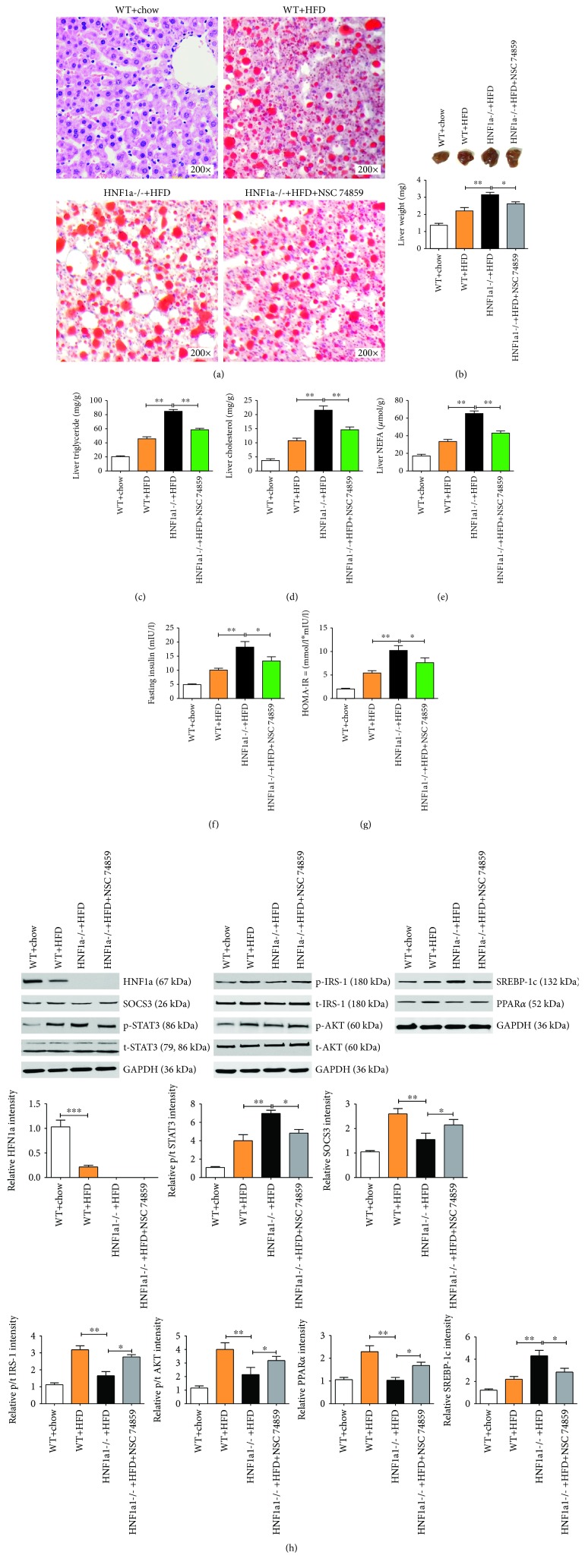
HNF1*α* inhibits steatosis through suppressing STAT3 *in vivo*. (a) Oil Red O staining showed that increased liver steatosis in HNF1*α*-/- mouse liver compared with WT HFD group. Treatment of NSC74859 in HNF1*α*-/- mice rescued them from severe steatosis. (b) Liver weight of HNF1*α*-/- mice were significantly higher than WT mice fed with HFD, treatment of NSC74859 decreased mouse liver weight. (c–e) Biochemical indicators showed that HNF1*α* deficiency increased triglyceride (TG), cholesterol (TC), and nonesterified fatty acid (NEFA) contents from the liver tissue. After treating the mice with NSC74859, the contents of TG, TC, and NEFA decreased significantly. (f, g) Serum fasting insulin levels were determined by ELISA, and homeostasis model assessment of insulin resistance (HOMA-IR) index was calculated as [FBG (mmol/l) × FIns (mIU/l)]/22.5. *n* = 4–8 per group, at the 8th week. HNF1*α* defect mice were significantly higher than WT mice fed with HFD. After treating the mice with NSC74859, the insulin levels and the HOMA-IR index decreased significantly. (h) Western blot analysis showed that HNF1*α* deficiency increased the expression of SREBP-1c and phosphorylation of STAT3 and reduced the expressions of SOCS3 and PPAR*α* and phosphorylation of IRS-1 and AKT. Treating HNF1*α* defect mice with NSC74859 reversed these protein expressions: NSC74859 inhibited the expression of SREBP-1c and promoted the expression of PPAR*α*. All values are expressed as mean ± SEM, *n* = 8–12 per group, ^∗^*p* < 0.05, ^∗∗^*p* < 0.01, and ^∗∗∗^*p* < 0.001.

To confirm our results found in LO2 cells, we adopted an *in vivo* model using HNF1*α* defect mice. Studies on HNF1*α* using different knockout models have been reported before [23–25]. Herein, we show that after 8 weeks of HFD feeding, HNF1*α*-/- mice had increased liver steatosis compared with WT group (Figure 7(a)). However, *in vivo* treatment of HNF1*α*-/- mice with NSC 74859 significantly reduced liver steatosis. This data suggests that HNF1*α* deficiency-induced liver steatosis is STAT3 dependent which is consistent with our results in LO2 cells as showed in Figure 6. Similarly, the liver sizes or weights were larger in HNF1*α*-/- mice while treating HNF1*α*-/- mice with NSC 74859 reduced both liver size and weight (Figure 7(b)). We also tested triglyceride, cholesterol, NEFA, fasting insulin, and HOMA-IR index; all these parameters were significantly higher in HNF1*α*-/- mice; and inhibition of STAT3 with NSC 74859 partially ameliorated these characters (Figures 7(c)–7(g)). Western blot analysis first confirmed the expression of HNF1*α* in liver tissue (Figure 7(h)). HNF1*α* deficiency led to decreased expressions of SOCS3 and PPAR*α* and phosphorylation of AKT and IRS-1 while phosphorylation of STAT3 and expression of SREBP-1c were increased in HNF1*α*-/- mouse liver cells (Figure 7(h)). Interestingly, *in vivo* treatment of HNF1*α*-/- mice with NSC74859 slightly increased phosphorylation of IRS-1 and AKT compared with HNF1*α*-/- mice (Figure 7(h)). We also showed that NSC74859 inhibited the expression of SREBP-1c and promoted the expression of PPAR*α*. NSC74859 reversed the effects of deficiency of HNF1*α* expression on SREBP-1c and PPAR*α* expression (Figure 7(h)). These data further confirm that HNF1*α* regulates hepatic lipid metabolism by promoting the expression of SOCS-3 and negatively regulating the STAT3 signaling pathway.

## 4. Discussion

Here, we evaluated the effect and clarified the regulation and mechanism of HNF1*α* on hepatic glycolipid metabolism. Our results showed that FFA-induced hepatocyte LO2 steatosis inhibited the expression of HNF1*α*. NAFLD, which is characterized by excessive deposition of hepatic fat, is extremely harmful, but its developmental mechanism is still unclear. Our results suggested that the development of NAFLD may be related to the excessive deposition of hepatocyte fat together with inhibition of HNF1*α* and other genes essential for maintaining normal liver function. The inhibition of HNF1*α* on FFA-induced hepatic LO2 steatosis also demonstrated that HNF1*α* is involved in the regulation of hepatic fat metabolism and has the effect of preventing excessive deposition of hepatic fat. SREBP-1c is an important transcription factor that regulates the *de novo* synthesis and glycolysis pathways of fat. SREBP-1c regulates fatty acid synthase and can cause lipid deposition, which plays an important role in the pathogenesis of NAFLD [26]. PPAR*α* is a transcription factor of the nuclear hormone receptor superfamily. Its main function in the liver is as a lipid sensor, recognizing fatty acids flowing into the liver and regulating the expression of specific genes affecting lipid metabolism [27]. PPAR*α* plays an important role in the oxidative decomposition of fat and has a protective effect on NAFLD [28, 29]. We found that HNF1*α* inhibits the expression of SREBP-1c and promotes the expression of PPAR*α*. This result further indicates that HNF1*α* is involved in the regulation of hepatic lipid metabolism. HNF4*α* has been reported to play a key role in controlling hepatic CES2 expression in diabetes, obesity, or NASH [30]; thus, we also investigated and found that HNF1*α* positively regulates CES2 expression though much need to be done in the future (data not shown). Mouse experiments have also confirmed that liver-specific knockdown of the HNF1*α* gene leads to increased fatty acid synthesis in the liver and excessive deposition of fat in the liver [31].

Genome-wide association analysis (GWAS) results revealed a correlation between the HNF1*α* mutations and the potential risk of developing type 2 diabetes [32]. Insulin resistance is the crucial initiating factor in the development of metabolic diseases such as NAFLD and diabetes. To achieve its function, insulin must first bind to the insulin receptor on the cell surface and phosphorylate to activate IRS. The activated IRS continues to activate PI3K/AKT, which regulates glucose transport and glycogen synthesis. Our results show that HNF1*α* promotes phosphorylation with IRS-1 and AKT, i.e., HNF1*α* promotes activation of the insulin signaling pathway. The regulation of lipid metabolism by HNF1*α* is a potential cause of its close association with the development of metabolic diseases such as NAFLD and type 2 diabetes.

Previous studies have shown that inhibition of liver HNF1*α* not only increases lipid synthesis and excessive fat deposition but also promotes phosphorylation of STAT3 [31, 33]. Our results also confirmed that HNF1*α* promotes the expression of SOCS3, which is a STAT3 signaling pathway negative feedback regulator, and inhibits STAT3 phosphorylation. That is, HNF1*α* inhibits the activation of the STAT3 signaling pathway by promoting the expression of SOCS3. Mouse experiments have shown that liver-specific knockdown of STAT3 increases hepatic glucose production [34]. In insulin-resistant diabetic mice, overexpression of STAT3 not only increases plasma triglyceride and total cholesterol levels but also promotes transcription of lipid synthesis-related enzymes such as fatty acid synthase and acetyl-CoA carboxylase [35]. This indicated that STAT3 was involved in the regulation of hepatic glycolipid metabolism and in maintenance of hepatic glycolipid homeostasis.

Our results indicated that HNF1*α* inhibits lipid anabolism, promotes lipolysis, and promotes the activation of the insulin signaling pathway. There is a close relationship between hepatic glucose metabolism and lipid metabolism, which share many common regulatory elements and metabolites. Combined with previous studies, we concluded that HNF1*α* achieved the above metabolic regulation through the STAT3 signaling pathway. To further confirm our inference, we used the STAT3 inhibitor NSC74859 after downregulating the HNF1*α* expression. The results showed that the STAT3 inhibitor NSC74859 can reverse the effect of HNF1*α* on glucose and lipid metabolism, supporting our inference.

## 5. Conclusions

In summary, HNF1*α* promotes the activation of insulin signaling pathways, encourages fat decomposition, and inhibits the metabolic regulation of lipid synthesis via negatively regulating the STAT3 signaling pathways. This result indicates that HNF1*α* is likely to prevent excessive deposition of hepatocyte fat by negatively regulating the STAT3 signaling pathway, thus forming a protective effect on NAFLD. This provides an effective strategy for the treatment of NAFLD, insulin resistance, and type 2 diabetes.

## Data Availability

The data used to support the findings of this study are available from the corresponding author upon reasonable request.
